# Novel Biomarkers for Atherosclerotic Disease: Advances in Cardiovascular Risk Assessment

**DOI:** 10.3390/life13081639

**Published:** 2023-07-27

**Authors:** Raul-Alexandru Jigoranu, Mihai Roca, Alexandru-Dan Costache, Ovidiu Mitu, Alexandru-Florinel Oancea, Radu-Stefan Miftode, Mihai Ștefan Cristian Haba, Eosefina Gina Botnariu, Alexandra Maștaleru, Radu-Sebastian Gavril, Bogdan-Andrei Trandabat, Sabina Ioana Chirica, Raluca Maria Haba, Maria Magdalena Leon, Irina-Iuliana Costache, Florin Mitu

**Affiliations:** 1 Department of Medical Specialties I, Faculty of Medicine, University of Medicine and Pharmacy “Grigore T. Popa”, 700115 Iasi, Romania; jigoranu_raul-alexandru@d.umfiasi.ro (R.-A.J.); ovidiu.mitu@umfiasi.ro (O.M.); oancea.alexandru-florinel@d.umfiasi.ro (A.-F.O.); radu-stefan.miftode@umfiasi.ro (R.-S.M.); mihai.haba@umfiasi.ro (M.Ș.C.H.); alexandra.mastaleru@gmail.com (A.M.); rgavril87@yahoo.com (R.-S.G.); leon_mariamagdalena@yahoo.com (M.M.L.); irina.costache@umfiasi.ro (I.-I.C.); mitu.florin@yahoo.com (F.M.); 2 Department of Cardiology, “St. Spiridon” Emergency County Hospital, 700111 Iasi, Romania; 3 Clinical Rehabilitation Hospital, 700661 Iasi, Romania; 4 Department of Internal Medicine II, Faculty of Medicine, University of Medicine and Pharmacy “Grigore T. Popa”, 700115 Iasi, Romania; eosefina.botnariu@umfiasi.ro; 5 Department of Diabetes, Nutrition and Metabolic Diseases, “St. Spiridon” Emergency County Hospital, 700111 Iasi, Romania; 6 Department of Surgery II, Faculty of Medicine, University of Medicine and Pharmacy “Grigore T. Popa”, 700115 Iasi, Romania; trandabat.b@gmail.com; 7 Department of Orthopedics and Trauma, Clinical Rehabilitation Hospital, 700661 Iasi, Romania; 8 Faculty of General Medicine, University of Medicine and Pharmacy “Grigore T. Popa”, 700115 Iasi, Romania; sabinahadimbu@gmail.com (S.I.C.); ralucavreme@yahoo.com (R.M.H.); 9 Romanian Academy of Medical Sciences, 030167 Bucharest, Romania; 10 Romanian Academy of Scientists, 050045 Bucharest, Romania

**Keywords:** atherosclerosis, apolipoprotein B, interleukin-6

## Abstract

Atherosclerosis is a significant health concern with a growing incidence worldwide. It is directly linked to an increased cardiovascular risk and to major adverse cardiovascular events, such as acute coronary syndromes. In this review, we try to assess the potential diagnostic role of biomarkers in the early identification of patients susceptible to the development of atherosclerosis and other adverse cardiovascular events. We have collected publications concerning already established parameters, such as low-density lipoprotein cholesterol (LDL-C), as well as newer markers, e.g., apolipoprotein B (apoB) and the ratio between apoB and apoA. Additionally, given the inflammatory nature of the development of atherosclerosis, high-sensitivity c-reactive protein (hs-CRP) or interleukin-6 (IL-6) are also discussed. Additionally, newer publications on other emerging components linked to atherosclerosis were considered in the context of patient evaluation. Apart from the already in-use markers (e.g., LDL-C), emerging research highlights the potential of newer molecules in optimizing the diagnosis of atherosclerotic disease in earlier stages. After further studies, they might be fully implemented in the screening protocols.

## 1. Introduction

Cardiovascular diseases (CVDs) continue to remain the foremost cause of mortality on a global scale, with coronary artery disease (CAD) emerging as the most prevalent among them [1]. In Europe, CVDs account for approximately 45% of the overall deaths. Furthermore, approximately 4 million individuals die annually due to cardiovascular (CV) events, resulting in a staggering loss of over 60 million years of life due to CVDs in Europe alone [2,3]. While historically, CVDs predominantly affected the elderly population, a shift in paradigm has occurred due to the increasing incidence of diabetes, obesity, and smoking, leading to a growing number of patients under the age of 65 being diagnosed with these pathologies. In light of this, enhancing the existing screening methods and discovering novel ones should be accorded the utmost priority by medical systems worldwide [4].

In general practice, we rely on standardized CV risk scores (SCORE diagram, U-prevent algorithm, ASCVD score) to identify classic CV risk factors such as age, gender, blood pressure, cholesterol levels, smoking status, and diabetes. Even though multiple studies proved their efficiency, it is noteworthy that 50% of the patients who develop CAD are previously classified in the low- or intermediate-risk group [5]. The latest guideline on Chronic Coronary Syndromes from the European Society of Cardiology (ESC) identifies several complementary risk-assessment tools, namely intima-media thickness, coronary artery calcium scores, brachial flow-mediated dilation, ankle-brachial index, high sensitivity C-reactive protein (hs-CRP) and family history of CAD, which may be used in selected patients. However, they recommend using these tools sparingly, due to a lack of scientific proof of their efficiency [6]. Considering this, it is natural to say that there is an urgent need to find alternative instruments able to improve the existing algorithms’ accuracy and give a higher discrimination power to the existing scores.

The United States National Institutes of Health defines biomarkers as “a characteristic that is objectively measured and evaluated as an indication of normal biological processes, pathogenic processes, or pharmacologic responses to a therapeutic intervention”. Even if, after 2010, an increased rate of their use has been registered, they are far from being fully implemented instruments in medical practice, this term dates from as far back as 1970. From infectious diseases and cancers to cardiovascular pathologies such as heart failure (HF), many molecules can serve as indicators of the systemic impact of a particular disease, providing valuable guidance to medical professionals [7]. However, based on our knowledge, the scientific evidence strongly supporting a specific biomarker is lacking when it comes to atherosclerosis and CV risk stratification. Therefore, our article aims to focus on presenting the latest evidence that supports the significance of two particular molecules, interleukin 6 (IL-6) and apolipoprotein B (apoB), as potential biomarkers for atherosclerosis. The article will primarily emphasize their molecular structure and elucidate their involvement in the pathogenesis of atherosclerosis.

Firstly, we will present the most recent studies concerning the pathogenesis of atherosclerosis in the human body, including its underlying processes and influential factors. Secondly, we will expand upon the subjects of low-density lipoproteins (LDL), apoB, hs-CRP, and IL-6. Finally, we will present the potential role of apoB and IL-6 as future screening biomarkers, together with other emerging components.

## 2. Pathophysiology of Atherosclerosis

Atherosclerosis serves as the underlying cause for various CV events and its manifestations, including CAD and myocardial infarction; peripheral artery disease (PAD) and lower limb amputation; and aortic aneurysm (AA), renal artery stenosis (RAS), and carotid artery stenosis (CAS) associated with strokes [8]. Due to its characteristic risk factors—elevated serum cholesterol, diabetes, smoking, male gender, age, obesity, and chronic systemic inflammation—atherosclerosis tends to be more prevalent in higher-income countries, with a continuous and steady rise in incidence [1].

From the pathophysiological point of view, endothelial dysfunction, hypercholesterolemia, and inflammation represent the central triad of atherosclerosis [8]. The endothelium, the internal layer of the vascular wall, is the interface between the bloodstream and the main site of atherosclerosis. Thus, its integrity is mandatory to prevent lipid accumulation within the arteries. However, phenotypic changes in the endothelium’s cellular structure may alter its protective capacity, opening the path to atherosclerosis, sheer stress playing a central role in this process [9]. Laminar shear stress, physiologically exerted by the bloodstream, has an atheroprotective effect. On the other hand, disturbed blood flow, commonly observed near branch sites, generates low shear stress (frictional drag), activating the endothelial cells, diminishing their vasodilative and anti-thrombotic properties, associating increased oxidative stress, and promoting lipid uptake beneath the endothelium [10]. Once activated, endothelial cells also facilitate atherosclerosis and lipid accumulation by promoting inflammation and intimal hyperplasia via adhesion molecules, cytokines, and growth factors [11]. Adhesion molecules, such as vascular cell adhesion protein 1, intercellular adhesion molecule 1, P-selectin, and E-selectin, bind the circulating inflammatory cells and keep them trapped within the arterial wall. Subsequently, the secreted cytokines and chemokines attract new inflammatory cells to the atherosclerotic site. Lastly, growth factors, including platelet-derived growth factor, determine extracellular matrix deposition and smooth muscle proliferation [8,12].

The effects exerted by the activated endothelium lead to the over-activation of inflammatory cells, primarily targeting the cholesterol deposits beneath the endothelium [13]. This excessive inflammatory state manifests not only at the local site but also systemically, as evidenced by literature documenting a direct correlation between atherosclerosis and specific inflammatory biomarkers [14]. Additionally, the latest guidelines from the ESC on Chronic Coronary Syndromes recommend the use of hs-CRP as a complementary biomarker for assessing atherosclerosis [6]. Thus, comprehending the role of inflammation in the pathophysiology of atherosclerosis is crucial for preventing and treating this condition. The main leukocytes recruited into the vascular wall are monocytes, which become macrophages under the influence of the locally secreted cytokines [15]. Despite their initial protective role in removing cytotoxic oxidized LDL (ox-LDL), which they bind to via scavenger receptors (SR-A1, CD36, and LOX1/SR-E1), macrophages become a central component of the atherosclerotic plaque. Due to their inability to effectively digest LDL, it tends to accumulate within the inflammatory cells, leading to the formation of foam cells [13,16]. Subsequent accumulation of these pathological macrophages leads to fatty streak formation, which is, from the morphopathological point of view, the foundation of the future atherosclerotic plaque. In a desperate attempt to eliminate the abnormal lipids, proinflammatory (IL-1, IL-6, IL-12, IL-15, IL-18) and anti-inflammatory cytokines (IL-10 and TGF-β) are secreted, and the inflammatory response is augmented [17]. Those macrophages that fail to eliminate their lipid content and undergo cell death by apoptosis via endoplasmic reticulum activation are eliminated by other macrophages via efferocytosis [18]. Subsequently, when this process becomes overwhelmed, the foam cells recruit new inflammatory pathways. Dendritic cells play an important role in initiating the adaptative immune response. They are responsible for atherosclerosis-related antigen presentation to T cells. The predominant type of T cells in the atherosclerotic plaque are the T-helper type 1 lymphocytes (Th1), also implicated in the inflammatory process associated with myocardial infarction and HF [19,20]. They are central regulators of the adaptative immune response, able to differentiate into other T-helper subtypes, having either pro- or anti-inflammatory effects [13]. Type B lymphocytes are also present in the atherosclerotic process, being represented by B1, IgM antibody-producing cells and B2, IgG antibody-producing cells [17]. Contrary to the former, which proved to have an anti-atherosclerotic effect mediated by IL-5, IgG antibodies are associated with an increase in the atherosclerotic process [21]. The supplementary activation of the inflammatory response generates new foam cells, which determine the secretion of more cytokines and reactive oxygen species, transforming atherosclerosis into a vicious circle [8,22].

Lipids represent the final, but certainly not the least significant element of the atherosclerotic triad. A proportionality relationship exists between atherosclerosis and serum lipid levels [8,23]. If, theoretically, every human being developed atherosclerosis at some point in their life, the extent of the process and the moment of its development would be tightly correlated to their cholesterolemia [24]. Within the bloodstream, cholesterol particles exist in different forms. However, at least for the general population, LDL represents the most atherogenic fraction. Serum levels of LDL-cholesterol (LDL-C) are not the only determinant of the degree of atherosclerosis. In order to become atherogenic, the lipid particles need to suffer different types of structural changes, such as desialylation, glycation, and oxidation, with native LDL not causing lipid accumulation in the arterial wall [23]. After entering the arterial wall, LDL is internalized by the macrophages and contributes to the vicious circle already presented [8,25].

**Figure 1:**
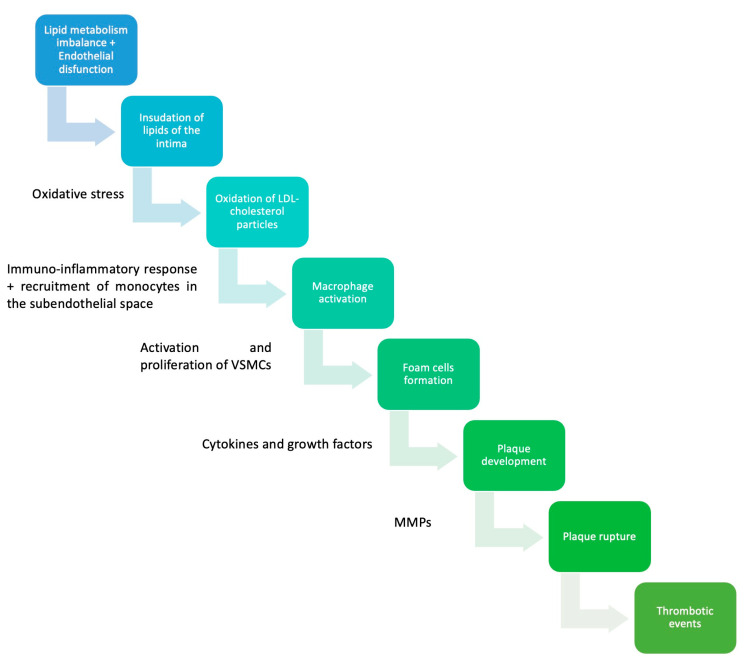
Pathophysiology of atherosclerosis. VSMCs—vascular smooth muscles cells; MMPs—matrix metalloproteinases [29,30,31].

As previously mentioned, the initial manifestation of atherosclerosis is the fatty streak, which develops very early in life, typically during adolescence or even childhood. For the atherosclerotic plaque to progress into a more advanced lesion, there must be an accumulation of extracellular lipids, forming extensive pools beneath the endothelium. These lipid pools serve as the core of the atheroma [26]. In response to inflammatory cytokines such as TNF-alpha, smooth muscle cells and fibroblasts from the outer layers of the arterial wall are recruited to the subendothelial site. Fibroblasts secrete large quantities of collagen, which forms the fibrous layer of the atheroma, giving rise to a new type of atherosclerotic lesion known as fibroatheroma [26,27]. Over time, the fibrous cap becomes weakened by locally secreted proteolytic enzymes, rendering it susceptible to rupture. This exposes the thrombogenic core to the bloodstream. From a clinical perspective, these events underlie some of the most concerning manifestations of atherosclerosis, including unstable angina, myocardial infarction, sudden cardiac death, and stroke [8,22,26]. While it was previously believed that cells producing the fibrous cap in the atherosclerotic plaque originated exclusively from smooth muscle cells, recent evidence has revealed that a significant portion of these cells are derived from endothelial cells and macrophages. This discovery has paved the way for identifying novel therapeutic approaches to enhance plaque stability (see Figure 1; see Table 1) [28].

## 3. LDL

LDL serves as the primary carrier of cholesterol and it is recognized as the most abundant atherogenic particle. Many existing therapies are targeted towards this molecule [32,33]. It possesses an average diameter of 22 nm and exhibits a dynamic molecular structure comprised of three layers: the core, which contains approximately 170 triglycerides and 1600 cholesteryl esters; the outer surface layer, primarily composed of phospholipids; and the interfacial layer, formed by the interpenetration of core and surface lipids [32]. Unesterified cholesterol molecules serve as a shared structural component in all three layers, with approximately one-third comprising the core and the remainder constituting the surface layer [34]. LDL has a complex proteome within its structure, apolipoprotein B 100 (apoB-100) being the primary protein in this lipoprotein [35]. It mediates the interactions between LDL and other molecules and receptors, being essential to all lipoprotein-related physiological processes. ApoB-100 accounts for approximately one-fifth of the weight of LDL and consists of three alpha-helical and two beta-structured domains. It has a molecular mass of 550kDa, being the largest monomeric glycoprotein known [36]. As shown by electron microscopy studies, apoB-100 wraps the surface of LDL molecules, forming a dense protein halo protecting the lipidic core and remaining attached to them throughout the entire lipid metabolism, given its hydrophobic character [32,37].

In order to determine LDL-C, multiple options are available. The first method, initially described in 1972, is the Friedewald formula. It estimates LDL-C using total cholesterol, high-density lipoprotein (HDL) cholesterol (HDL-C), and triglycerides (TGs). This formula presents a series of limitations, mainly imposed by TG levels, as in patients with TG > 250mg/dl it becomes inaccurate [38]. A response to the Friedewald’s formula’s limitations has appeared: the Martin equation. Contrary to what we have previously presented, in Martin’s equation, TGs are divided by an adjustable factor according to plasma TGs and nonHDL-C levels. However, neither the Martin equation nor the Friedewald formula are validated for TG > 400mg/dl. A new equation using more advanced and accurate mathematical instruments was introduced recently. We refer to the Sampson equation, which may be used for TG levels up to 800 mg/dl; however, not all studies support its superiority over the Martin equation [39]. LDL-C may also be determined using direct assays, which measure and do not estimate LDL-C. These methods are also called homogenous assays, as, in order to count LDL-C, they do not separate LDL from other lipoproteins but rather consume or mask them. They also impose some limitations, as alterations in the composition and size of lipoproteins decrease these methods’ accuracy [38]. A more recently introduced LDL quantification method uses nuclear magnetic resonance spectrometry to quantify the number and also the size of LDL particles [40].

### 3.1. Structure-Based Alterations of LDL

While the serum level of LDL-C serves as an important marker of atherogenicity, recent studies have revealed that native LDL does not readily accumulate within the arterial wall. For lipid molecules to become atherogenic, they need to suffer different phenotypical changes that facilitate their entry through the endothelial layer and promote the atherosclerotic process [23]. The existing evidence in the literature indicates that desialylation might be the earliest modification suffered by the native LDL, which promotes its atherogenicity. Desialylated LDL accounts for as much as 60% of the total circulating LDL in patients suffering from CAD [41]. In the molecular structure of LDL, sialic acid is an essential component of apoB, as it represents the terminal carbohydrate of the biantennary sugar chains. Removing sialic acid leads to multiple structural and functional alterations in the LDL particle, including size reduction, increased density, negative electric charge, loss of lipids, and oxidation. These changes collectively contribute to the heightened atherogenicity of desialylated LDL [42,43]. Desialylated LDL may be detected in human serum using a lectin-sorbent assay. However, there are few data supporting the use of this molecule as a biomarker for atherosclerosis [44]. On the other hand, we found a study cited in the literature suggesting the potential utility of desialylated apoB-100, detected using an ELISA assay, in predicting the presence of carotid atherosclerosis [45].

In addition to desialylation, glycation is another extensively studied structural modification of LDL that contributes to its increased atherogenicity [23]. Glycation is a non-enzymatic reaction between glucose and the exposed lysine residues of proteins. While it primarily occurs in proteins, it can also affect lipoproteins, LDL being particularly rich in glycated apoB-100 [46]. Similar to desialylation, glycated LDL is smaller, facilitating its accumulation within the arterial wall. It also has a slower catabolic rate, and due to apoB’s structural alteration, it is not adequately cleared by the LDL receptors [23,47]. Besides the structural changes, recent studies showed that glycated LDL may increase atherosclerotic risk by increasing platelet aggregation. The precise mechanism underlying this effect is not fully understood, but it may be linked to inhibiting the thrombocytes’ membrane Na^+^/K^+^ channels and the concomitant stimulation of Ca^2+^ ATP-ase, which raises intracellular calcium levels, increasing the response of platelets to ADP [48]. Glycated LDL can be measured using ELISA assay. However, this is not a routine technique in daily practice, and the literature lacks data supporting its use as a biomarker of atherosclerosis [49].

Lastly, oxidation is another essential reaction for the atherosclerotic process [50]. Ox-LDL is a strong promoter of atherosclerosis, as it is a major component of foam cells [51]. Compared to native LDL, ox-LDL has a higher affinity for scavenger receptors, making it more readily internalized by macrophages and promoting foam cell formation [52]. Moreover, ox-LDL possesses several biological properties that further contribute to atherosclerosis, including chemotactic activity for monocytes, promotion of growth factor expression, cytotoxic effects, endothelial dysfunction, and stimulation of platelet adhesion. Oxidation of LDL occurs primarily within the arterial wall by resident vascular cells and macrophages [52]. In vitro studies showed that LDL suffers some degree of oxidation during glycation, even in the absence of oxygen free radicals and oxygen-radical-generating processes [47]. Although ox-LDL levels in plasma are generally low due to plasma’s antioxidant properties, advances in ELISA techniques have enabled the detection of these particles with high sensitivity [53,54]. These advances led to a growing interest in researching ox-LDL as a biomarker of CVD. In a meta-analysis, which included 12 studies, increased circulating levels of ox-LDL proved to be associated with an increased risk of CV events [55]. In a prospective study, the ox-LDL/LDL ratio was positively associated with the severity of CAD, assessed using the Gensini score [56]. Additionally, increased levels of ox-LDL were also observed in patients with carotid artery disease. The hypothesis stating that ox-LDL may be a better indicator of the total CV risk is plausible, considering the pathophysiological implications of this molecule [57].

### 3.2. Lipid-Lowering Therapies and CV Risk

Statins represent the most prescribed drugs used to control the CV risk. They represent the first-line agents used to lower LDL-C. Their efficiency in reducing the rate of cardiovascular events, both as primary and secondary prevention, made them the cornerstone of CV risk management in guidelines [58]. Relatively recent medical research has led to the development of more potent lipid-lowering therapies such as proprotein convertase subtilisin/kexin type 9 (PCSK9) inhibitors. By regulating the number of LDL receptors responsible for LDL degradation, PCSK9 indirectly influences LDL-C levels. Lower levels of PCSK9 are associated with an increase in LDL receptor density and, consequently, a reduction in LDL-C [59]. Even if the efficiency of these molecules in reducing LDL-C has been proven by numerous randomized controlled studies, whether their effects go beyond cholesterol lowering remains unclear [60,61,62].

HUYGENS is a randomized controlled trial which tested the effects of Evolocumab on atherosclerotic plaques. The study included 161 patients who underwent coronary angiogram for NSTEMI and presented at least one angiographic lesion in addition to the culprit plaque, which implied a stenosis <50%. Other inclusion criteria which concerned the atherosclerotic plaques were having at least one image of fibrous cap thickness ≤120µm and lipidic ac >90°. Patients were randomly assigned to the placebo group or to the Evolocumab group, receiving 420mg/month for 48 weeks. For the atherosclerotic plaque study, optical coherence tomography (OCT) and intravascular ultrasound (IVUS) examination was performed at the baseline and after 50 weeks. At the end of the follow-up period, patients receiving Evolocumab presented a significantly better plaque regression, reflected by fibrous cap thickening (+39.0 µm vs. +22.0 µm, *p* = 0.015) and lipid arc decrease (−57.5° vs. −31.4°, *p* = 0.04). Additionally, an interesting finding was that in the Evolocumab group, the length of the vessel containing macrophages was reduced significantly (−3.17 mm vs. −1.45 mm, *p* = 0.04) [63].

PACMAN-AMI is a similar trial that tried to evaluate the effects on atherosclerotic plaques of the early administration of PCSK9 inhibitors in patients suffering from acute myocardial infarction. This study was centered on Alirocumab and included 300 randomly allocated participants to receive 150mg of Alirocumab or placebo for 52 weeks. To evaluate the effects of Alirocumab at the end of the follow-up period, they used: the percentage of atheroma volume (PAV), determined using IVUS; fibrous cap thickness, measured using OCT; and lipid core burden evaluated using near-infrared spectroscopy. Patients who received the PCSK9 inhibitor showed a greater reduction in PAV compared to placebo (−2.13% vs. −0.92%, *p* < 0.001). Similar changes were also evident for lipid burden index reduction (−79.42 vs. −37.6, *p* = 0.006) and fibrous cap thickness (+62.67 µm vs. 33.19 µm, *p* = 0.001) [64].

PCSK9 inhibitors represent an important step forward when it comes to dyslipidemia treatment and CV risk management, mainly due to their efficiency in LDL-C reduction. Based on their findings regarding the effects of Alirocumab and Evolocumab on plaque stability, HUYGENS and PACMAN-AMI bring us closer to understanding what lies behind the efficiency of these molecules [63,64].

## 4. Apolipoprotein B

### 4.1. Structure and Secretion

ApoB is a protein possessing amphipathic properties, serving as a structural framework for all lipoproteins implicated in atherogenesis. These lipoproteins include very low-density lipoproteins (VLDL), intermediate-density lipoproteins (IDL), LDL, and chylomicrons [65]. ApoB exists in two primary forms: apoB-100, a full-length apolipoprotein comprising of 4536 amino acids, predominantly found in VLDL and its metabolic derivatives, and apoB-48, which is synthesized by the intestine and consists of only 2152 amino acids, playing a key role in chylomicrons [66].

ApoB-100 is synthesized by the liver, depending on lipid availability [67]. Contrary to other secretory proteins, apoB-100 becomes exposed to the cytosol of the endoplasmic reticulum very early in the post-translational period. This allows its rapid proteasomal degradation via chaperon protein binding immunoglobulin [68], the liver secretion of apoB-100 being easily regulated proportionally to the need for lipoprotein synthesis. Eventually, apoB-100 is maturated in the Golgi apparatus, where VLDL is formed [69,70].

Structurally, apoB-100 comprises five α-helical structures and β sheet domains, closed by one N-terminal region and another C-terminal one. Each of these structures has specific functions [71]. The βα1 N-terminal domain is a binding site for microsomal triglyceride transfer protein (MTP). MTP is a specific apoB protein needed for VLDL assembly and for scavenger receptors [72]. The β1 domain is essential for lipid binding and lipoprotein formation, being the anchor that facilitates the firm adhesion of apoB-100 to the surface of different lipoproteins [73]. Another important binding site is the β2 domain, located at the C-terminal portion, assuring the interaction between apoB-100 and LDL receptors [74]. Lastly, α2 and C-terminal α3 domains are responsible for core lipid recruitment, forming the flexible region of the apolipoprotein [75].

### 4.2. Role as a Biomarker

Most lipid-lowering and cardiovascular risk-management protocols currently in use focus primarily on targeting LDL [76]. This approach proves effective for a significant proportion of patients, as achieving the recommended LDL-C levels helps reduce the risk of fatal CV events. However, in certain situations, despite reaching the therapeutic goals, these events may not be prevented, suggesting the existence of a residual atherogenic risk. This residual risk may be related to triglyceride concentrations and cholesterol content within particles rich in triglycerides [67,77]. Thus, the need for an alternative biomarker efficient in these particular situations is evident. As measurement methods for apoB have become more and more accessible, research interest in this molecule has considerably increased, and so has its potential as an atherosclerosis biomarker. A 2011 meta-analysis that incorporated data from 12 epidemiological studies aimed to compare the predictive capabilities of LDL-C, non-HDL cholesterol, and apoB in relation to fatal and non-fatal CV events. The analysis utilized the relative risk ratio (RRR) as the measure of predictive power. The study’s findings indicated that among the three investigated risk factors, LDL-C exhibited the weakest association, with a mean RRR of 1.25, while apoB emerged as the strongest indicator of CV risk, with a mean RRR of 1.34. Furthermore, in direct comparison, the RRR for apoB was found to be 5.7% higher than that of non-HDL cholesterol (95% CI, 2.4% to 9.1%; *p* < 0.001) and 12% higher than LDL-C (95% CI, 8.5% to 15.4%; *p* < 0.0001) [78]. Another representative research that evaluated the association of apoB with the total CV risk is AMORIS. This prospective study included 175,553 patients, who were followed up for a mean period of 66.8 months. The study aimed to assess whether apoB adds any predictive power regarding CV events to the classic cholesterol profile. Similarly to the meta-analysis previously presented, apoB not only increased the predictive power of LDL-C when added to statistical models, but it was also a better predictor of the CV risk in individuals with low LDL-C [79]. As a response to the growing data supporting the use of apoB as a CV risk-assessment tool, the latest ESC guidelines for the management of dyslipidemias recommend routine measurement of apoB for patients suffering from pathologies such as hypertriglyceridemia, diabetes, obesity, and metabolic syndrome [76]. 

Furthermore, the atherogenicity of lipids is influenced by their mass, with smaller particles having a greater propensity to penetrate the arterial wall. Consequently, LDL-C, which measures the total mass of cholesterol contained in LDL particles, may not accurately reflect the CV risk [65]. On the other hand, apoB reflects a number of a wider spectrum of atherogenic particles but does not provide information about their mass. By combining these two biomarkers using the LDL-C/apoB ratio, valuable insights can be gained regarding the average mass of circulating atherosclerotic particles. This information may prove useful in assessing an individual’s CV risk [80]. A retrospective case-cohort study, which included 1058 diabetic patients, aimed to evaluate the association between a low LDL-C/apoB ratio and the presence of coronary heart disease (CHD). Participants were included in the CHD group if they had a positive history of percutaneous coronary intervention, coronary bypass graft, acute coronary syndrome, or myocardial infarction. The study concluded that patients with CHD had a significantly lower LDL-C/apoB ratio (*p* = 0.001). Moreover, an LDL-C/apoB ratio ≤ 1.2 was able to predict the presence of CHD, independently of the ASCVD score (OR = 1.841, *p* = 0.002) [81]. More importantly, Jung et al. found in their study that the LDL-C/apoB ratio was an important predictor of significant coronary artery stenosis (>50%) and the need for revascularization. Their research included non-diabetic patients, extending the use of this biomarker outside the borders specified in the ESC guidelines. However, in their study, apoB alone did not offer any additional predictive power for coronary stenosis over LDL-C [82].

Contrary to apoB, apolipoprotein A-I (apoA-I) plays a protective role in the atherosclerotic process. It is the main component of HDL, and increased serum levels of apoA-I have proven to be associated with a lower risk of CV events [83,84]. Provided that each of these two biomarkers give separate information regarding the atherogenicity of one’s plasma, several studies attempted to combine their predictive power under the apoB/apoA-I ratio. Liting et al. conducted a prospective study which included over 820 patients with coronary angiography indications. ApoB, apoA-I, and the apoB/apoA-I ratio were measured for each study participant. Statistical analysis revealed that patients with angiographically significant coronary lesions (>50%) had higher levels of apoB and apoB/apoA-I ratio and lower apoA-I levels. Furthermore, when CAD patients were divided into groups according to the severity of their lesions, they observed that the apoB/apoA-I ratio was proportional to the extension of the coronary disease. At the end of the 3-year follow-up period, they also concluded that the apoB/apoA-I ratio better predicted long-term CV events than LDL-C [85].

Lastly, apoB proved to be a more powerful predictor for myocardial infarction than LDL-C and triglycerides. In a prospective study, which included over 430,000 patients, split into two groups (the primary and the secondary prevention group), apoB was the only independent predictor of the risk of myocardial infarction. Additionally, they tested whether the type of lipoprotein (triglyceride-rich lipoproteins or LDL) had any prognostic importance. They evaluated the triglyceride/LDL-C ratio while adjusting for apoB and clinical risk factors and concluded that the type of apoB-containing lipoprotein brought no additional risk-assessment value [86].

## 5. Interleukin 6

High hs-CRP has proved to be a promising predictor of future CV events in clinical trials conducted worldwide. However, the data supporting it as a routine biomarker for atherosclerosis are insufficient. IL-6 is a central mediator of acute phase response and an essential stimulant for hepatic CRP synthesis [87]. Going “upstream” in the pathophysiological chain of atherosclerosis-related inflammation, IL-6 plays a central role, along with IL-1, in this process. This relationship is supported by genetic research as well. Compared to hs-CRP, IL-6 has a shorter half-life and within-person variability, which limited the research regarding this molecule [88]. However, an increase in both molecules may be a better indicator of a chronic inflammatory process, as atherosclerosis is [89]. Having this in mind, recent studies support its utility for CV risk assessment [90,91]. Structurally, IL-6 is a phosphorylated glycoprotein consisting of four-helix bundles, which form a single chain. The four helices run in different directions; thus, helices A and B have opposite distributions than helices C and D. The linking between these components is made via four loops, connecting the bundles two by two [92].

Regarding its physiological effects, IL-6 is a ubiquitarian molecule secreted by various tissues and cells. In response to infection, trauma, or stress, IL-6 is expressed by monocytes and macrophages. The adipose tissue is another important source of IL-6, the latter being associated with obesity and diabetes. For different neoplastic tissues (myeloma, plasmacytoma, renal cell carcinoma, Kaposi sarcoma), IL-6 serves as a growth factor [93,94,95,96]. Studies in the literature also link IL-6 to the endothelial dysfunction associated with angiotensin-II-induced hypertension [97,98,99]. 

On the other hand, a potential protective effect of this interleukin was observed in studies conducted on IL-6 depleted mice. Schieffer et al. observed its atheroprotective role, as the deficit of IL-6 not only did not stop plaque formation but was associated with a more pronounced atherosclerotic process in their study [100]. Furthermore, Al-Khalili et al. observed that mice with IL-6 deficiency who developed obesity had a positive glucose and lipid metabolism response after short-term administration of IL-6. This might be explained by the fact that at the level of skeletal muscle, IL-6 ensures short-term energy supply and glucose mobilization [101,102]. However, to our knowledge, these results have yet to be reproduced in human subjects. Additionally, patients suffering from autoimmune diseases characterized by increased IL-6 levels, such as rheumatoid arthritis, tend to have a higher risk of developing CVD, supporting the role of IL-6 as a risk factor for atherosclerosis [103]. 

IL-6 plays its roles via a cell-surface receptor complex formed by a ligand-binding glycoprotein (IL-6R) and a signal-transducing component (gp130). IL-6R is an alpha-chain containing three domains. There are two types of IL-6R, the membrane-bound one (mIL-6R), mainly expressed in hepatocytes, neutrophils, and monocytes, and a soluble one (sIL-6R), which acts as a transporter for IL-6 [92].

### 5.1. IL-6 and the Risk of CV Events

The relationship between IL-6 and atherosclerosis is bidirectional. On the one hand, an intense atherosclerotic process is associated with increased IL-6 production due to inflammation. On the other hand, increased IL-6 levels, determined by extrinsic factors such as obesity, social stress, smoking, and pollution, may increase the atherosclerotic risk, as it is associated with increased insulin resistance, hypertension, dyslipidemia, and endothelial dysfunction [104]. Papadopoulos et al. observed in their meta-analysis, which included 11 population-based prospective cohort studies, a linear association between IL-6 levels and the risk of ischemic stroke. For each standard deviation increase in log-IL-6 levels, the risk of ischemic stroke increased by 19%. Their observation was independent of conventional CV risk factors and comparable to other ischemic stroke risk factors (non-HDL cholesterol and blood pressure) [105].

In another meta-analysis, which included eleven epidemiological studies and over 288,738 patients, IL-6 emerged as a predictive factor for CVD. The mean follow-up period was seven years. IL-6 baseline levels were associated with an increased risk of developing coronary heart disease at the end of the follow-up period. Furthermore, IL-6 levels correlated with the incidence of hypertension and hypercholesterolemia [106]. A prospective study conducted over 12 years, which included 1592 patients suffering from peripheral artery disease, showed a linear correlation between CRP, IL-6, and ICAM-1 and the disease progression, reflected by the ankle-brachial index (ABI) evolution. In the same study, IL-6 was the only biomarker able to predict the ABI decline at both 5 and 12 years, showing its superiority over the other molecules [107].

### 5.2. IL-6 and CAD

The existing literature provides supporting evidence for utilizing IL-6 as an indicator for subclinical CAD. In their prospective study, which included over 300 diabetic subjects, who underwent electron-beam cardiac computed tomography, Saremi et al. observed a significant association (*p* < 0.01) between IL-6 levels and the severity of CAD, reflected in the calcium score (CAC score). This association remained significant even after the adjustment for other risk factors. This type of relationship was not evident for CRP or Lp-PLA2 and CAC score [108].

Gerin et al. conducted a prospective study over 118 subjects who underwent coronary angiography. The analysis excluded those patients suffering from pathologies that could be associated with increased IL-6 levels. CAD disease was defined as the presence of at least one coronary stenosis >50%. CAD severity was quantified using SYNTAX and GENSINI scores. IL-6 levels correlated with the two severity scores, and they were higher in the CAD group. Furthermore, after the regression analysis, IL-6 proved to be an independent predictor for the presence of CAD, with serum levels >7.91 pg/mL being able to predict significative atherosclerotic plaques with a sensitivity of 78% and a specificity of over 70% [109]. Similar results were found in another recent prospective study, which included 1796 patients who underwent computed tomography angiography. Besides the association between IL-6 levels and the severity of CAD, serum levels >1.8ng/L were associated with an increased risk of death, myocardial infarction, or unstable angina. Additionally, in patients with non-obstructive coronary plaques (<70%), high-sensitivity troponin and IL-6 levels above the median were predictive for major adverse cardiovascular events [110].

These findings have been validated by other prospective and observational studies as well. However, in order to establish a consensus regarding the appropriate cut-off values for IL-6, further research is required [111,112,113].

### 5.3. Anti-inflammatory Therapy and CV Risk

The existing therapies designed to reduce the CV burden induced by atherosclerotic disease almost exclusively concentrate on cholesterol targets. Even though the inflammation hypothesis of atherothrombosis has been validated by numerous animal-based, epidemiological, and genetic studies, the causality effect remains to be established by future interventional trials. This is why there is a growing interest in exploring the efficiency of anti-inflammatory therapies in reducing CV risk [114]. A recent randomized, double-blind trial (CANTOS) assessed whether Canakinumab, a human monoclonal antibody targeting IL-1β, could reduce the total CV risk without affecting lipid levels. The study separated their cohort of over 10,000 patients into two groups: the placebo group or the Canakinumab group. The primary endpoints were nonfatal myocardial infarction, stroke, or cardiovascular death. The mean follow-up period was 3.7 years. At the end of this period, CRP and IL-6 were significantly reduced in the Canakinumab group. Additionally, compared to placebo, Canakinumab reduced the risk of the primary endpoints by 15% without influencing the lipid levels of the patients (3.86 vs. 4.5 events per 100 person-years) [115]. 

The fact that IL-1 is a major inducer of IL-6 production, as well as the effects of Canakinumab on IL-6 levels identified in the prior study, prompted the additional investigation. In an IL-6-centered analysis, Ridker et al. observed that the baseline levels of this cytokine were correlated with the risk of future CV events. More importantly, an on-treatment IL-6 level of <1.65 ng/L was associated with a reduction in the risk of major CV events of 32% (*p* < 0.0001) and a 52% reduction in CV mortality (*p* < 0.0001). Based on these findings, they concluded that there is a strong correlation between the IL-6 signaling pathway and the risk of CV events. Additionally, this study gives solid clinical arguments for the inflammatory hypothesis of atherothrombosis, supporting, at the same time, the use of IL-6 as a potential biomarker for the CV risk [116].

RESCUE is another recent randomized trial which tested the effects of Ziltivekimab, an antibody-mediated IL-6 inhibitor explicitly developed for atherosclerosis. This study included 264 participants with high CV risk, moderate to severe chronic kidney disease (CKD), and hs-CRP > 2 mg/L. They were randomly assigned to the placebo group or to Ziltivekimab at ascending doses (7.5 mg, 15 mg, 30 mg). At the end of the 24-week follow-up period, hs-CRP was reduced proportionally to the Ziltivekimab dose (77%, 88%, 92%), compared to the 4% reduction in the placebo group. Compared to Canakinumab from the CANTOS trial, Ziltivekimab was almost twice as efficient in reducing hs-CRP levels [117]. 

In response to the findings from the RESCUE and CANTOS trials, the concept of a new randomized, long-term endpoint trial called ZEUS emerged. The first results from this trial are expected in 2025, and it is meant to compare Ziltivekimab to a placebo among 6200 patients with moderate to severe CKD and elevated hs-CRP. The central question of this trial is whether reducing IL-6 levels can reduce CV event rates, testing one more time the causal effect of inflammation in atherothrombosis [118].

## 6. Role of Emerging Biomarkers of Atherosclerosis

Searching the existing literature, we found numerous other molecules currently under research which may act as biomarkers of atherosclerosis and CVD [51,119] (see Table 2).

Arginine vasopressin (AVP) is a pituitary gland peptide which was proven to be related to CV risk factors such as abdominal obesity and diabetes [120]. Copeptin is the C-terminal fragment of the precursor pre-provasopressin, which is released in the same amount as AVP, but has greater plasma stability [121]. There is a growing interest in researching this molecule and its relationship to the CV risk; however, most of the existing data are based on diabetic patients. Copeptin levels were associated with an increased risk of CAD, HF, and death in this population [122]. It was also proven to be a good indicator of subclinical atherosclerosis as it was also correlated with arterial stiffness [123]. Recently, similar results were obtained in non-diabetic patients. Tasevska et al. concluded that higher copeptin levels were predictive for CAD development, even though the association was stronger for non-diabetics. As for the risk of CV mortality, the relationship with copeptin was equally strong in both groups [124].

Midkine (MK) is a low molecular weight protein, representing the family of growth factors and cytokines. It is associated with both inflammatory and reparative processes [125]. Numerous studies have shown its relationship with CVD [126,127]. In atherosclerosis, MK is responsible for various pathophysiological processes, such as inflammation, neointimal formation, angiogenesis, and foam cell formation [125,128]. In a study conducted on patients referred for peripheral artery revascularization, higher levels of MK were observed in the diseased group compared to controls. Furthermore, as part of a diagnosis panel, MK proved to be efficient in predicting the presence of obstructive peripheral artery disease [129]. Similar results were obtained with a different panel tested for CAD [130]. 

Myeloperoxidase (MPO) is an inflammation-related enzyme, mainly secreted by phagocytes (monocytes, macrophages, and neutrophils). Its implication in the atherosclerotic process is related to LDL oxidation, a process that MPO supports mainly via oxidant-hypochlorous acid secretion [131]. Additionally, it was found that MPO is also a strong promoter of endothelial dysfunction, being responsible for nitric oxide bioavailability impairment [132]. A recent prospective study found a significant inverse correlation between MPO and brachial flow-mediated dilation, a measure of endothelial function. In the same study, higher levels of MPO were associated with a higher prevalence of subclinical carotid atherosclerosis [133]. In another prospective study, MPO proved to be a strong predictor for developing CAD (*p* = 0.001). The analysis included over 1100 patients, who were followed up for a mean period of 8 years [134]. 

Growth differentiation factor-15 (GDF-15) is a cytokine from the transforming growth factor-β superfamily [135]. It is expressed by various cells, including myocardial cells, adipocytes, macrophages, endothelial cells, and vascular smooth muscle cells. However, its serum levels are generally low in healthy individuals [136]. As a biomarker of CVD, GDF-15 proved its efficiency, especially in HF patients, for whom it appeared to be a reliable predictor of mortality [137] and disease severity [138]. As for atherosclerosis, its biological functions are not completely understood. However, GDF-15 seems to support this process by promoting inflammation and angiogenesis [139]. In patients suffering from acute coronary syndromes (ACS), GDF-15 has been demonstrated to be a powerful predictor of death [140,141] and risk of hospitalization for HF [142]. In stable CAD patients, GDF-15 is associated with CV and non-CV death [143] as well as with the risk of developing myocardial infarction [144]. In a retrospective study based on the Framingham Offspring Study cohort, GDF-15 levels were predictive for subclinical carotid atherosclerosis [145]. Further studies assessed the association between GDF-15 and CAD. A small prospective study concluded that increased GDF-15 levels were predictive for the presence of CAD, evaluated angiographically. In the same study, GDF-15 correlated with the Gensini score [146]. A positive correlation was also observed between GDF-15 and the extent of coronary collateral formation, described using the Rentrop grading system, which further linked it with the severity of CAD [147]. 

Lipoprotein-associated phospholipase A_2_ (Lp-PLA2) is an enzyme secreted especially by monocytes and macrophages [148]. It has a high specificity for vascular inflammation, and it circulates in the plasma primarily associated with LDL [149,150]. Lp-PLA2 promotes atherosclerosis by hydrolyzing the oxidized phospholipids in the LDL, which generates bioactive products, such as lysophosphatidylcholine, oxidized fatty acids, and arachidonic acid, capable of recruiting monocytes, activating endothelial cells, and stimulating smooth muscle cell proliferation [151,152]. The quantification of Lp-PLA2 can be performed using two methods: measuring its mass concentration using an ELISA immunoassay or assessing its enzymatic activity using a spectrophotometric assay. However, the latter proved to be more accurate [153]. In stable CAD patients, Lp-PLA2 activity proved to be a predictor of CV events, such as myocardial infarction, CV death, and stroke [154]. In another prospective study, Lp-PLA2 was positively correlated with the presence and the severity of CAD, independently of other CV risk factors (*p* < 0.05) [155]. Additionally, adding Lp-PLA2 to a biomarker panel, along with hs-CRP, increased the predictive power for atherosclerotic carotid disease, assessed using ultrasonography [156].

Using ultracentrifugation, LDL particles can be separated into four subclasses based on their density: subclass I—large LDL, subclass II—intermediate LDL, and subclasses III and IV, generally assigned as small dense LDL (sdLDL). Small dense LDL is the smallest and also the densest subclass, measuring less than 25.5nm and with a density that ranges from 1.034 g/mL to 1.060 g/mL [157,158]. Experimental studies suggested that compared to other LDL subfractions, sdLDL presents higher atherogenic properties due to several metabolic and structural features, including smaller size, which promotes endothelial penetration [158], a higher affinity for proteoglycans in the arterial wall [159], a lower affinity for LDL receptors [160], and higher susceptibility for oxidation, due to vitamin E deficiency [161]. Based on these results, the idea of using sdLDL as a biomarker for atherosclerosis and CV risk emerged. In a prospective cohort study, which included over 11,000 participants, sdLDL-C was associated with incident CHD (hazard ratio of 1.51, 95% CI, 1.21–1.88). Additionally, sdLDL-C was higher in patients with diabetes [162]. In another prospective trial conducted on patients who suffered an acute ischemic stroke, sdLDL was able to predict short-term mortality (*p* < 0.05) and also ischemic stroke onset (OR = 4.31, *p* < 0.001) [163]. This subfraction of LDL was also associated with coronary artery stenosis. In a small case-control study, which included over 190 patients referred for elective coronary angiography, sdLDL-C levels were predictive for the presence of significant coronary stenosis [164]. Additionally, there are studies in the literature, which link sdLDL-C levels to the risk of plaque progression and destabilization, strengthening the use of sdLDL as a marker for CV risk [165,166].

## 7. The Potential Use of Apolipoprotein B and Interleukin-6 as Future Screening Biomarkers for Cardiovascular Risk

**Figure 2:**
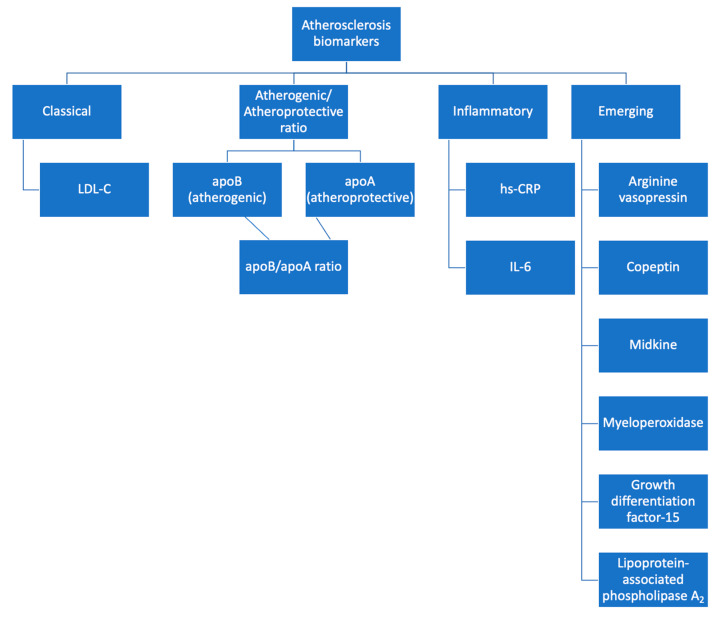
Biomarkers for atherosclerosis detection.

This review aimed to explore the pathophysiological basis that supports the role of these two molecules as potential biomarkers of atherosclerosis. Based on our research, there is a rising number of studies supporting the notion that apoB and IL-6 are able to predict both the risk of future CV events and the presence and severity of CAD. However, further prospective studies are needed in order for these molecules to be implemented in clinical practice (see Figure 2).

At the moment, LDL-C represents the only routine biomarker used by the guidelines to estimate the total CV risk, despite its limitations, especially in specific populations. However, based on the current existing data, apoB, the common piece that links all the atherosclerotic lipoproteins, might be a potential substituent for LDL-C, able to cross its boundaries [154].

Furthermore, inflammation plays a key role in the atherosclerotic process, and represents an important risk factor for CV events. Despite this, no inflammatory biomarker is currently used for the CV risk assessment, except for hs-CRP, whose utility is controversial. IL-6 is a strong stimulator of hs-CRP liver secretion and a central inflammation mediator in the atherosclerotic process, making it a good candidate for this position. Moreover, the rising number of prospective studies linking IL-6 serum levels to the severity of CAD supports future research on this topic [108,109,110,111,112,113].

These emerging biomarkers could prove useful in assessing individuals during the early stages of the atherosclerotic process when the cardiovascular risk is still low, thus enabling the faster initiation of the preventive measures before the onset of events such as CAD or stroke. This could also allow us to reclassify the patients based on the cardiovascular risk. However, at the moment, their sensibility and specificity should still be studied in larger groups, in order to fully understand their mechanisms, what influences their levels, and how exactly they correlate to the atherosclerotic process [108,109,110,111,112,113].

## 8. Conclusions

LDL-C, the classical and most routinely used parameter measured in patients, remains a viable marker of atherosclerosis and cardiovascular risk. More specific would be the measuring of apoB, which includes a wider spectrum of atherogenic particles. On the other hand, apoA is associated with an atheroprotective effect. Therefore, the ratio between the two would be an even more accurate assessment tool. Additionally, given the inflammatory nature of atherosclerosis, hs-CRP and IL-6 levels are indicative. This review offers a comprehensive perspective on the diagnosis of atherosclerosis through the analysis of both established and emerging biomarkers, with a potential to be fully implemented in future screening protocols.

## Figures and Tables

**Table 1:** Factors that contribute to atherosclerosis formation.

Monocyte Chemotaxis and Adhesion	Activation and Proliferation of VSMCs	Plaque Development	Plaque Instability /Rupture
ICAM-1 VCAM-1 MCP-1	TNF-α IFN-γ PDGF TGF-β bFGF	Cytokines Growth factors	MMPs

ICAM-1—intercellular adhesion molecule-1; VCAM-1—vascular cell adhesion molecule-1; MCP-1—monocyte chemoattractant protein-1; TNF-α—tumor necrosis factor-α; IFN-γ—interferon-γ; PDGF—platelet derived growth factor; TGF-β—transforming growth factor β; bFGF—basic fibroblastic growth factor [29,30,31].

**Table 2:** Emerging biomarkers of atherosclerosis.

Biomarker	Relation	References
Arginine vasopressin	abdominal obesity, diabetes	[120,121,122,123,124,125,126,127,128,129,130,131,132,133,134,135,136,137,138,139,140,141,142,143,144,145,146,147,148,149,150,151,152,153,154,155,156]
Copeptin	an increased risk of CAD, HF, arterial stiffness, subclinical atherosclerosis	
Midkine	inflammatory and reparative processes	
Myeloperoxidase	LDL oxidation	
Growth differentiation factor-15	heart failure	
Lipoprotein-associated phospholipase A_2_	vascular inflammation, LDL specificity	

## Data Availability

Not applicable.
